# Soluble *N*-Ethylmaleimide-Sensitive Factor Attachment Protein Receptor-Derived Peptides for Regulation of Mast Cell Degranulation

**DOI:** 10.3389/fimmu.2018.00725

**Published:** 2018-04-11

**Authors:** Yoosoo Yang, Byoungjae Kong, Younghoon Jung, Joon-Bum Park, Jung-Mi Oh, Jaesung Hwang, Jae Youl Cho, Dae-Hyuk Kweon

**Affiliations:** ^1^Biomedical Research Institute, Korea Institute of Science and Technology (KIST), Seoul, South Korea; ^2^Division for Bio-Medical Science & Technology, KIST School, Korea University of Science and Technology, Daejeon, South Korea; ^3^Department of Integrative Biotechnology, College of Biotechnology and Bioengineering, Sungkyunkwan University, Suwon, South Korea; ^4^Biomedical Institute for Convergence, Sungkyunkwan University, Suwon, South Korea; ^5^Department of Genetic Engineering, College of Life Science, Kyung Hee University, Yongin, South Korea

**Keywords:** soluble *N*-ethylmaleimide-sensitive factor attachment protein receptor, membrane fusion, mast cell, peptide, atopy, degranulation

## Abstract

Vesicle-associated V-soluble *N*-ethylmaleimide-sensitive factor attachment protein receptor (SNARE) proteins and target membrane-associated T-SNAREs (syntaxin 4 and SNAP-23) assemble into a core *trans*-SNARE complex that mediates membrane fusion during mast cell degranulation. This complex plays pivotal roles at various stages of exocytosis from the initial priming step to fusion pore opening and expansion, finally resulting in the release of the vesicle contents. In this study, peptides with the sequences of various SNARE motifs were investigated for their potential inhibitory effects against SNARE complex formation and mast cell degranulation. The peptides with the sequences of the N-terminal regions of vesicle-associated membrane protein 2 (VAMP2) and VAMP8 were found to reduce mast cell degranulation by inhibiting SNARE complex formation. The fusion of protein transduction domains to the N-terminal of each peptide enabled the internalization of the fusion peptides into the cells equally as efficiently as cell permeabilization by streptolysin-O without any loss of their inhibitory activities. Distinct subsets of mast cell granules could be selectively regulated by the N-terminal-mimicking peptides derived from VAMP2 and VAMP8, and they effectively decreased the symptoms of atopic dermatitis in mouse models. These results suggest that the cell membrane fusion machinery may represent a therapeutic target for atopic dermatitis.

## Introduction

Atopic dermatitis is a type of chronic skin disease that is associated with allergic inflammation. The immediate allergic reaction is caused by the uncontrolled degranulation of mast cells, which are specialized granulated cells that are localized to specific tissues and release various proinflammatory mediators, chemotactic factors, and immunoregulatory cytokines (1). Upon the stimulation of the surface receptor FcεRI with crosslinked preformed IgE (2), the degranulation of mast cells is activated, and subsequently, their content of proinflammatory mediators is released *via* membrane fusion between preformed granules/vesicles and the outer cell membrane (3, 4).

The processes of vesicular transport, fusion, and the release of vesicle contents, including those occurring during mast cell degranulation, are mediated by the fusion machinery proteins known as the soluble *N*-ethylmaleimide-sensitive factor attachment protein receptor (SNARE) proteins in all eukaryotic cells (3). SNARE proteins are classified as vesicle-localized (V)-SNAREs and target-localized (T)-SNAREs depending on their cellular localization. Both types of SNAREs mediate membrane fusion by forming a highly stable SNARE complex comprising four conserved SNARE motifs (5).

The commercial success of the botulinum neurotoxin (BoNT), which controls neuronal exocytosis and muscle contraction, explicitly exemplifies that SNARE proteins are versatile therapeutic targets (6, 7). Various serotypes of BoNT specifically cleave individual SNARE proteins. In contrast, it has long been conceived that the inhibition of SNARE complex formation may also result in BoNT-like activity (8–12) because four SNARE motifs from three or four SNARE proteins work together after forming the SNARE complex (13, 14). The design of competitive inhibitors of SNARE complex formation may provide an avenue for the development of SNARE-specific drugs. A straightforward way to design such competitive inhibitors is to use peptides that have the amino acid sequences of SNARE motifs. The peptides that compete efficiently with the assembly of the native SNARE complex are likely to strongly inhibit the function that the SNARE complex performs. For example, synthetic peptides containing fewer than 20 residues patterned after the N- or C-terminal region of SNAP-25 have been reported to inhibit neuronal SNARE complex formation and neurotransmitter release (15–17). In a previous study, we also showed that peptides derived from the N-terminal region of SNAREs potently blocked neuroexocytosis. Those peptides efficiently inhibited SNARE complex formation in neuronal cells and blocked neurotransmitter release equally as effectively as verapamil, which is an L-type calcium channel blocker (9).

As several SNARE proteins responsible for mast cell degranulation have been identified, it should be possible to use the same approach as neuroinhibitory peptides to prevent allergic reactions. Mast cells use the synaptosome-associated protein of 23 kDa (SNAP-23 or SN23) and syntaxin 4 (Syn4) as T-SNAREs localized on the plasma membrane during IgE-dependent degranulation, while several mRNAs encoding V-SNAREs including vesicle-associated membrane protein 2 (VAMP2), VAMP3, VAMP7, and VAMP8 have been reported to be expressed in rat basophilic leukemia (RBL-2H3) mast cells (18–20). In our previous work, we also measured the mRNA expression of various SNARE isoforms including VAMP2, VAMP4, VAMP7, and VAMP8 (4). However, it was shown that the binary complex of SNAP-23 and Syn4 could functionally mediate membrane fusion with only VAMP2 or VAMP8, but not with VAMP4 or VAMP7 (4, 8, 21). Based on the validation of functional ternary SNARE complexes, seventeen 17-mer peptides derived from the sequences of mast cell SNARE motifs were synthesized. The inhibitory efficacies of those peptides against mast cell degranulation and atopic dermatitis were evaluated. The inhibition of SNARE complex formation *in vitro* and *in vivo* by the peptides was also measured. Among the synthetic peptides, those having sequences derived from the N-terminal regions of SNARE motifs were the most efficient in reducing the degranulation of RBL-2H3 cells, *in vitro* membrane fusion, and SNARE complex formation in mast cells. The N-terminal conjugation of a protein transduction domain (PTD) to the synthetic peptides enhanced the *in vitro* transduction of SNARE peptides without any alteration of their inhibitory activities (22). In particular, the lead peptides Vp2N and Vp8N significantly relieved the atopic dermatitis symptoms of NC/Nga mice, indicating that these peptides have great potential as drugs for the treatment of atopic dermatitis.

## Materials and Methods

### Cloning, Expression, and Purification of Recombinant SNARE Proteins

Total RNA was obtained from RBL-2H3 cells using TRIzol^®^ Reagent (#15596026, Invitrogen, Carlsbad, CA, USA) and served as a template for cDNA synthesis with M-MLV reverse transcriptase (M1705, Promega, Madison, WI, USA) according to the manufacturer’s protocol. The cDNAs amplified for rat Syn4, SNAP-23, VAMP2, VAMP4, VAMP7, and VAMP8 using each primer pair (4), were cloned into the pGEX-4T 1 vector. The mast cell SNARE proteins [Syn4 (amino acids 1–298), SNAP-23 (amino acids 1–210), full-length VAMP2 (VpF, amino acids 1–116), VAMP4 (amino acids 1–141), and VAMP8 (amino acids 1–100)] were expressed in *Escherichia coli* Rosetta(DE3)pLysS (#70956, Novagen, Darmstadt, Germany) and purified as glutathione-*S*-transferase-tagged fusion proteins as reported previously (8).

Briefly, the *E. coli* cells were grown at 37°C in Luria–Bertani medium (#244610, BD Biosciences, San Diego, CA, USA) and the expression of the recombinant protein was induced by treating the cells with 0.3 mM isopropyl β-d-thiogalactoside when their optical density at 600 nm reached 0.8. Then, the cells were harvested by centrifugation at 8,000 *g* for 10 min and lysed by sonication (45% amplitude, 1.5 min net sonication, 1 s on–1 s off). The lysate was clarified by centrifugation (10,000 *g*, 20 min, 4°C), and bound to glutathione agarose beads (G4510, Sigma-Aldrich, St. Louis, MO, USA). Following a thorough washing step, the glutathione-*S*-transferase-tagged SNAREs were cleaved using thrombin (27-0846-01, GE Healthcare, Pittsburgh, PA, USA). The purity of all proteins, as assessed by sodium dodecyl sulfate-polyacrylamide gel electrophoresis using 12.5% polyacrylamide gels, was greater than 90%.

### Mast Cell Culture and Degranulation Assay

RBL-2H3 cells purchased from the American Type Culture Collection (CRL-2256), were grown in high-glucose Dulbecco’s modified Eagle’s medium supplemented with 10% heat-inactivated fetal bovine serum, 100 U/mL penicillin G, and 100 µg/mL streptomycin at 37°C in an atmosphere of 5% CO_2_. Degranulation assays to determine the amount of released β-hexosaminidase or histamine in the supernatant were performed as described previously (23, 24). Briefly, RBL-2H3 mast cells, seeded into 24-well plates (2 × 10^5^ cells/well), were washed with 4-(2-hydroxyethyl)-1-piperazineethanesulfonic acid (HEPES)-buffered saline [140 mM NaCl, 5 mM KCl, 1 mM CaCl_2_, 0.6 mM MgCl_2_, 0.1% glucose, 0.1% bovine serum albumin (BSA), and 10 mM HEPES] and then treated with 100 ng/mL anti-dinitrophenyl (DNP)-IgE (D8406, Sigma-Aldrich) for 3 h to sensitize them for antigen-induced degranulation. The cells were further incubated with or without 10 µM peptide inhibitors. The PTD-conjugated peptide inhibitors were applied to the cells alone, whereas the non-conjugated peptide inhibitors were applied together with 10 U streptolysin O (SLO) (S5265, Sigma-Aldrich) to permeabilize the cell membrane (25). After 30 min, 200 ng/mL 2,4-dinitrophenylated BSA (DNP-BSA) or 10 µM A23187 was applied to the cells for antigen-induced or ionophore-induced degranulation, respectively. Degranulation was evaluated in HEPES-buffered saline for 30 min at 37°C and quantified by measuring β-hexosaminidase or histamine release using colorimetric biochemical assays. For the measurement of β-hexosaminidase release, 50-µL aliquots of the cell culture medium supernatant were mixed and incubated with 50 µL substrate solution (1 mM *p*-nitrophenyl-*N*-acetyl-β-D-glucosaminide in 100 mM citrate, pH 4.5) for 3 h at 37°C in a 96-well plate.

Following the addition of 100 µL NaCO_3_–NaHCO_3_ buffer (1:1 mixture, 100 mM NaCO_3_–NaHCO_3_, pH 10.0) to terminate the reaction, the amount of β-hexosaminidase released was determined by measuring the absorbance at 405 nm using a microplate reader. These measurements were then normalized to the total β-hexosaminidase content of the cells, which was determined by dissolving the cells with 0.1% Triton X-100™. The cell culture medium supernatants were also used to measure the release of histamine using a histamine enzyme immunoassay kit (Oxford Biomedical Research, Rochester Hills, MI, USA) according to the manufacturer’s protocol. The percentage release of β-hexosaminidase or histamine was calculated as follows (26): Release (%) = [(*T* − *B* − *N*)/(*C* − *N*)] × 100, where *T* (test) is the absorbance value in the presence of both DNP-BSA and drugs [DNP-BSA (+) and drugs (+)], *B* (blank) is the absorbance value of DNP-BSA (−) and drugs (+), *C* (control) is the absorbance value of DNP-BSA (+) and drugs (−), and N (normal) is the absorbance value of DNP-BSA (−) and drugs (−).

### Preparation of Proteoliposomes and SNARE-Driven Membrane Fusion Assay

To prepare the unilamellar liposomes, a 50-mM lipid mixture of palmitoyl-2-oleoylphosphatidylcholine (POPC) (#850457, Avanti Polar Lipids, Alabaster, AL, USA):1,2-dioleoyl-phosphatidylserine (DOPS) (#840035, Avanti Polar Lipids) at 65:35 mol% was dried in a glass tube under a gentle stream of nitrogen gas and then exposed to a vacuum overnight. The resultant lipid film was resuspended in dialysis buffer (25 mM HEPES and 100 mM KCl, pH 7.4) by vortexing, followed by an extrusion step with polycarbonate membrane filters having a pore size of 100 nm (#610005, Avanti Polar Lipids). To prepare proteoliposomes, binary T-SNAREs, which were pre-formed by mixing Syn4 and SNAP-23 at room temperature for 60 min, were incubated with 50 mM unilamellar liposomes for 15 min at the same temperature, resulting in a 50:1 lipid/protein molar ratio (9).

For the preparation of V-SNARE proteoliposomes, a 10-mM premixed lipid solution [POPC:DOPS:*N*-(7-nitro-2-1,3-benzoxadiazol-4-yl (NBD))-phosphatidylserine (#810198, Avanti Polar Lipids):rhodamine-phycoerythrin (#810150, Avanti Polar Lipids) = 62:35:1.5:1.5 (molar ratio)] was used for the preparation of fluorescent liposomes in the same manner as above. The prepared liposomes were then mixed with VAMP2 for 15 min at room temperature. The liposome/protein mixture was diluted twice to bring the concentration of *n*-octyl glucoside (O8001, Sigma-Aldrich) below the critical micelle concentration. After overnight dialysis against 1L dialysis buffer at 4°C to remove the detergent, SM-2 bio-beads (#1523920, Bio-Rad Laboratories, Hercules, CA, USA) were added to the sample to remove any remaining *n*-octyl glucoside. The solution was then centrifuged at 10,000 × *g* to remove the protein/lipid aggregates, and the final lipid concentrations of both the T- and V-liposomes were adjusted to 1 mM with a lipid-to-protein ratio of 50:1.

A total lipid mixing assay using the T- and V-liposomes was performed as described previously with minor modifications (8). Briefly, peptide inhibitors were applied to the T-vesicles at the indicated concentrations and then the T-vesicles were mixed with the V-vesicles in a 384-well plate for the initiation of fusion, with a T- to V-vesicle volume ratio of 9:1. The fusion was monitored using the dequenching of NBD fluorescence signal (excitation 465 nm/emission 530 nm). After at least 80 min of fusion, the maximum NBD signal was obtained by adding 0.1% Triton X-100™. All the lipid-mixing experiments were performed at 37°C.

### Co-Immunoprecipitation and Immunoblotting Analysis

Lysates of RBL-2H3 cells (0.5–1 × 10^6^ cells), which were obtained by the addition of 1 mL lysis buffer (50 mM Tris–HCl, 150 mM NaCl, 1% Triton X-100™, 1 mM EDTA, pH 7.5) supplemented with a protease inhibitor cocktail (Calbiochem^®^, Merck Millipore, Billerica, MA, USA), were centrifuged at 13,000 *g* at 4°C to remove cell debris and precleared with 100 µL protein G Sepharose^®^ (GE Healthcare) for 2 h at 4°C. The precleared lysates were incubated with antibodies against SNARE proteins (Syn4, SNAP-23, VAMP2, and VAMP8) for 2 h at 4°C for binding, and then the antibody–antigen complexes were precipitated by incubation with a protein G slurry overnight with constant shaking. After an extensive washing step with lysis buffer, an immunoblotting analysis was performed for the precipitates. The immunoprecipitated proteins and cell lysates were electrophoresed on 12% sodium dodecyl sulfate-polyacrylamide gel electrophoresis gels and then transferred to nitrocellulose blotting membranes (GE Healthcare). After blocking the membranes in TBST (10 mM Tris–HCl, 150 mM NaCl, and 0.05% Tween^®^ 20, pH 8) containing 0.5% BSA and 5% skim milk for 1 h at room temperature, primary anti-SNAP-23 antibody (1:1,000 dilution) in TBST supplemented with 1% BSA and 0.01% sodium azide was added and incubated at 4°C overnight. The membranes were washed with TBST, treated with a horseradish peroxidase-conjugated secondary anti-mouse IgG antibody (A4416, Sigma-Aldrich), and visualized using enhanced chemiluminescence solution for the development of a luminescent signal.

### Animal Model of Atopic Dermatitis

Seven-week-old NC/Nga mice were purchased from the Shizuoka Laboratory Animal Center (Shizuoka, Japan) and allowed to adapt to the environment for a week prior to use. The procedures for the induction of atopic dermatitis-like symptoms in mice using 1-chloro-2,4-dinitro-benzene (DNCB) have been well described previously (27). Briefly, the dorsal hair of the mice was shaved 2 days before DNCB administration. For the induction of atopic dermatitis, 1% DNCB was applied to the dorsal skin of the mice twice weekly for 7 days and then 0.2% DNCB was applied thrice weekly for 21 days. The mice were divided into the following seven treatment groups (*n* = 10 per group): 0.3 and 3% Vp2N/day, 0.3 and 3% Vp8N/day, 0.1% dexamethasone/day, and vehicle only, dorsally administered from 9 to 13 weeks of age. As a negative control for atopic dermatitis, NC/Nga mice that were not treated with DNCB were maintained under pathogen-free conditions. Laboratory animal breeding management was based on the “Guide for the Care and Use of Laboratory Animals,” and all experiments were approved by the Institutional Animal Care and Use Committees of Gyeonggi Institute of Science & Technology.

### Statistical Analysis

The statistical analyses were performed using Student’s *t*-test using Sigma Plot 10.0 software to determine the *p* value (*p*-values < 0.05 were considered statistically significant and individual *p*-values are shown in figure legends). Values are expressed as mean ± SD for control and experimental samples.

## Results

### Peptides Having the Sequences of Various SNARE Motifs

**Figure 1:**
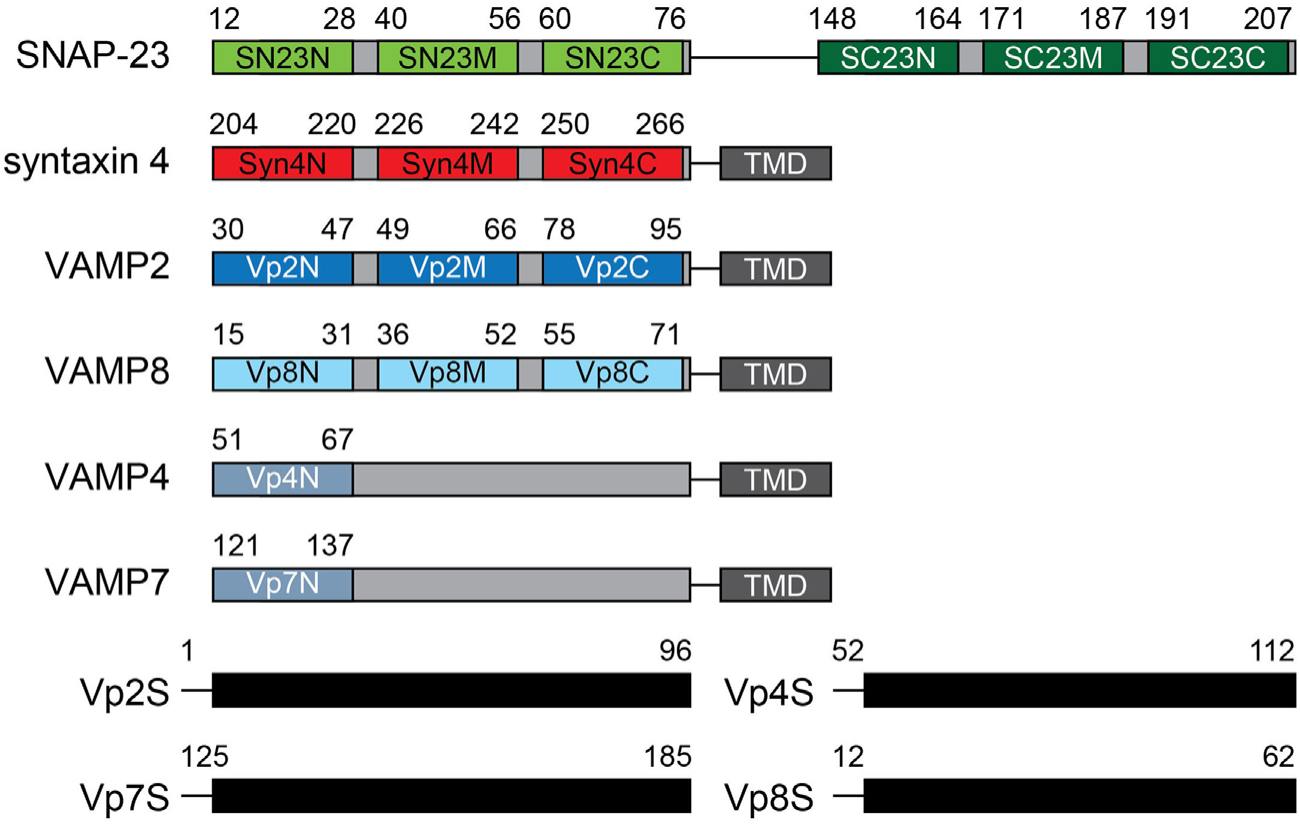
Design of mast cell soluble *N*-ethylmaleimide-sensitive factor attachment protein receptor (SNARE) motif-patterned peptides. Peptides patterned after the α-helical regions of the SNARE motifs of SN23 (green), syntaxin 4 (red), and VAMPs (blue) were designed. The names of the peptides are shown in the motif diagram, and the residue number in the motif corresponding to each peptide is indicated above each diagram. The amino acid sequences of the synthetic peptides are shown in Table 1. The soluble domains of various VAMP proteins are also indicated. TMD, transmembrane domain.

To search for potent peptide inhibitors that can interfere with SNARE complex formation and thereby reduce mast cell degranulation, seventeen 17-mer peptides were synthesized of which sequences were patterned after the N-terminal (N), middle (M), and C-terminal (C) regions of the SNARE motifs of SNAP-23 (SN23 and SC23), Syn4, and VAMPs (Vp2, 4, 7, and 8) (Table 1; Figure 1). Those four SNARE proteins were selected as the template sequences because the activity of their ternary complexes was validated in previous studies using the lipid mixing assay (4, 8, 21). Additionally, the full-length soluble SNARE motifs of VAMP2, VAMP4, VAMP7, and VAMP8, which are known to be present in mast cell granules, were expressed and purified from *E. coli*. The PTDs were fused to the N-terminal regions of the synthetic peptides to provide the peptides with the ability to cross cell membranes (22). PTDs are small peptides that are used to carry proteins, peptides, nucleic acids, nanoparticles, and other types of cargo across the plasma membrane into cells (28–30). Shorter peptides with six or eight amino acid residues were not tested because they did not inhibit membrane fusion by neuronal SNARE proteins even at 200 µM concentration (9).

**Table 1:** Amino acid sequences of synthetic peptides derived from mast cell soluble *N*-ethylmaleimide-sensitive factor attachment protein receptor (SNARE) motifs.

Name	Sequence
SN23N	RAHQVTDESLESTRRIL
SN23M	KTITMLDEQGEQLNRIE
SN23C	DQINKDMREAEKTLTEL
SC23N	EDEMEENLTQVGSILGN
SC23M	DMGNEIDAQNQQIQKIT
SC23C	DTNKNRIDIANTRAKKL
Syn4N	SARHSEIQQLERTIREL
Syn4M	FLATEVEMQGEMINRIE
Syn4C	DYVERGQEHVKIALENQ
Vp2N	RRLQQTQAQVDEVVDIM
Vp2M	VNVDKVLERDQKLSELD
Vp2C	QFETSAAKLKRKYWWKN
Vp8N	LQSEVEGVKNIMTQNVE
Vp8M	RGENLDHLRNKTEDLEA
Vp8C	EHFKTTSQKVARKFWWK
Vp4N	KIKHVQNQVDEVIDVMQ
Vp7N	QSLDRVTETQAQVDELK
Protein transduction domain (PTD)	YARVRRRGPRRGGG

*Seventeen 17-mer peptides representing the N-terminal, middle, and C-terminal regions of the SNARE motifs of SN23, syntaxin 4, Vp2, Vp8, Vp4, and Vp7 were synthesized. PTDs were added to the N-terminus of each peptide to facilitate their penetration into cells*.

### Selective Inhibition of Degranulation in RBL-2H3 Mast Cells by Synthetic Peptides

**Figure 2:**
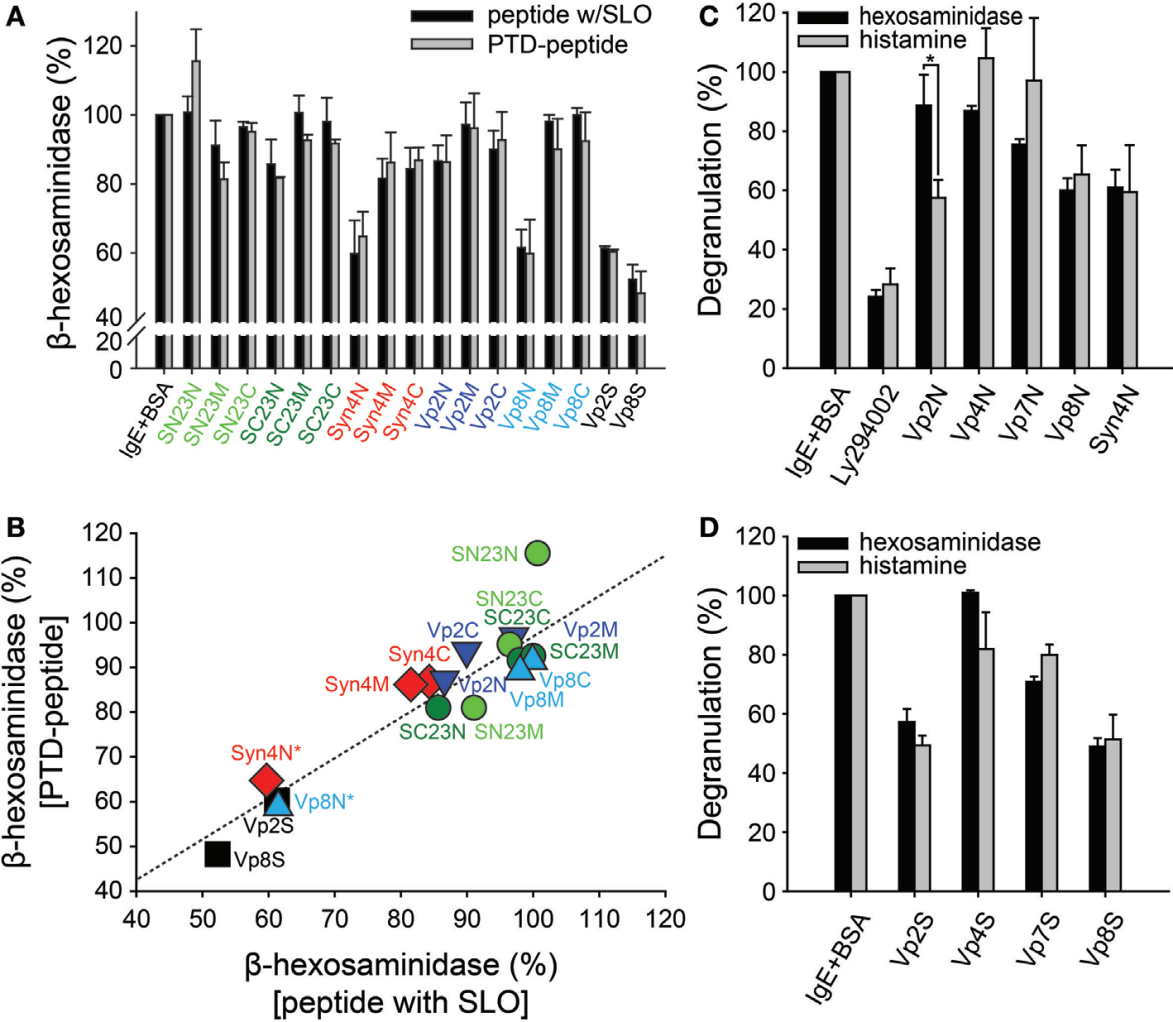
Effect of synthetic peptides on the degranulation of RBL-2H3 mast cells. **(A)** RBL-2H3 mast cells grown on 24-well plates were treated with dinitrophenyl (DNP)-specific IgE and then exposed to the synthetic peptides (10 µM) together with streptolysin-O (SLO). After incubation for 30 min, the cells were stimulated with 2,4-dinitrophenylated bovine serum albumin (DNP-BSA) for degranulation. For comparison, protein transduction domain (PTD)-conjugated synthetic peptides were tested in the same manner as described above without SLO treatment. The soluble domains of vesicle-associated membrane protein 2 and 8 (Vp2S and Vp8S) were included in the experiment as positive controls. **(B)** Correlation between the results of degranulation from the synthetic peptides with SLO and the PTD-conjugated peptides without SLO. **(C,D)** The inhibitory effects of the synthetic peptides on the mast cell degranulation of distinct granule subsets. Mast cells sensitized with DNP-IgE were treated with PTD-conjugated peptides **(C)** or soluble proteins **(D)**, followed by stimulation with DNP-BSA. Cell culture medium supernatants were assayed for the detection of degranulation. LY294002 was used as a positive control inhibitor of FcεRI-mediated degranulation in mast cells. All data are presented as the mean ± SD from three independent experiments. The statistical significance of differences was evaluated by analysis of variance (*p < 0.05).

Mast cells, including RBL-2H3 cells, possess three distinct types of secretory granules: type I amine-free granules containing β-hexosaminidase, type II granules containing both β-hexosaminidase and histamine, and type III granules containing secretary amines (31, 32). Therefore, the degranulation of mast cells has typically been measured by examining the release of granule mediators, especially β-hexosaminidase and histamine (33, 34). To investigate the effect of synthetic peptides on mast cell degranulation, sensitized RBL-2H3 cells were treated with 10 µM synthetic peptides to inhibit SNARE complex formation together with the bacteria-derived toxin SLO to temporarily permeabilize the cell membranes (35). After incubation at 37°C for 30 min for the uptake of the peptides into the RBL-2H3 cells, degranulation was stimulated with DNP-BSA. After harvesting the culture medium, the activity of β-hexosaminidase was measured *via* a colorimetric biochemical assay. Vp2S and Vp8S reduced β-hexosaminidase release from mast cells by 40–50% (Figure 2A, black bars). While most of the tested peptides were ineffective, Syn4N and Vp8N also reduced β-hexosaminidase release by 30–40%.

The cells were treated with 10 µM PTD-fused peptides without SLO treatment. Consistently with the previous results, Syn4N and Vp8N reduced β-hexosaminidase release from mast cells by 30–40% (Figure 2A, gray bars). The other tested peptides did not show significant inhibitory activities against β-hexosaminidase release. These results suggested that PTD not only efficiently transduced SNARE-derived peptides without SLO but also did not disrupt the inhibitory activity of the peptides. As such, the inhibition of β-hexosaminidase degranulation by both treatment methods (peptide + SLO or PTD-fused peptide) showed a strong linear relationship (Figure 2B).

It is remarkable that the inhibitory activity of Vp2N was specifically observed against histamine release (Figure 2C). Vp2N did not inhibit the release of β-hexosaminidase, but it inhibited the release of histamine from mast cells by 43%. In contrast, Vp8N and Syn4N almost equally inhibited the release of both β-hexosaminidase and histamine (Figure 2C). The inhibitory effects of Vp4N and Vp7N were in the low range of 0–20%. This result suggested that the inhibition of mast cell degranulation by Vp2N peptides might be specific to type III granules.

Previous studies have shown that mast cells possess distinct secretory granule subsets whose exocytosis is regulated by different SNARE isoforms (4, 36). Moreover, it was also shown that VAMP8 is characterized as a V-SNARE connected to type I and type II granules, whereas VAMP2 is only involved in the trafficking of amine-containing type III granules (19). However, granule selectivity was not observed from the soluble domains of the SNARE motifs of Vp2S, Vp4S, Vp7S, and Vp8S. While Vp2S and Vp8S, which are involved in mast cell degranulation, were more potent inhibitors of degranulation than Vp4S and Vp7S, none of all four of the full-length SNARE motifs showed selectivity toward β-hexosaminidase and histamine (Figure 2D).

### Selective Inhibition of SNARE-Mediated Membrane Fusion by N-Terminal-Mimicking Peptides

**Figure 3:**
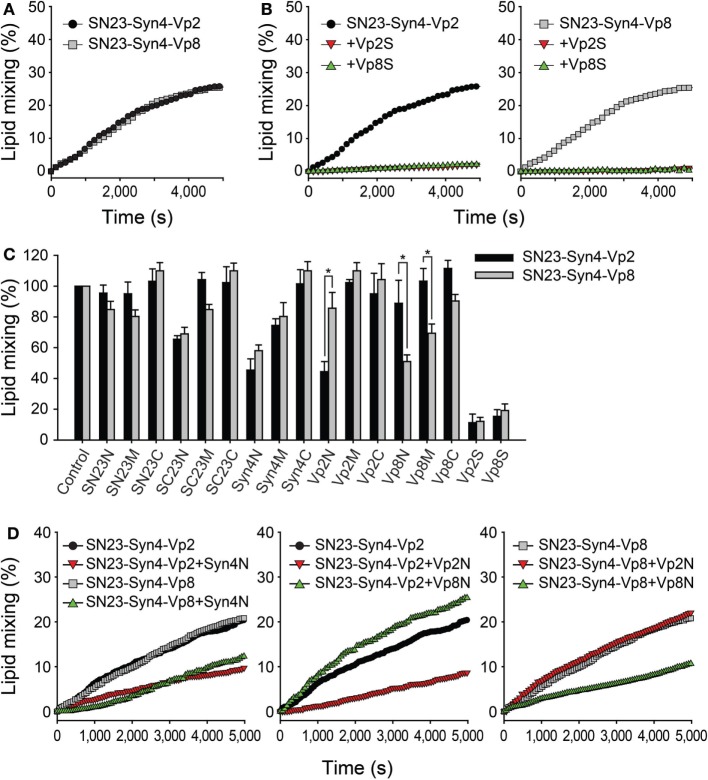
Inhibitory effects of the synthetic peptides on SNARE-driven membrane fusion. **(A)** Lipid mixing assay of T-liposomes containing syntaxin 4 (Syn4) and SN23 together with V-liposomes prepared with Vp2 (black) or Vp8 (gray). The assay was monitored by measuring the dequenching of NBD fluorescence and the data were plotted as a function of time. The maximum NBD signal was obtained by adding 0.1% Triton X-100™. **(B)** The soluble N-ethylmaleimide-sensitive factor attachment protein receptor (SNARE)-mediated membrane fusion was completely blocked by the cytoplasmic domains of Vp2 (Vp2S, red) and Vp8 (Vp8S, green) at the concentration of 10 µM. **(C)** The inhibitory effects of synthetic peptides on lipid mixing using mast cell SNAREs including Vp2 (black) and Vp8 (gray). Each bar represents the mean ± SD of three independent experiments. The statistical significance of differences was evaluated by analysis of variance (*p < 0.05). **(D)** The curves represent the lipid mixing driven by SN23-Syn4-Vp2 or SN23-Syn4-Vp8 in the presence of the most effective peptides (Syn4N, Vp2N, and Vp8N).

A lipid-mixing assay was performed using SNARE-reconstituted liposomes (37, 38). Proteoliposomes containing either V-SNARE (Vp2 or Vp8) and both T-SNAREs (SN23 and Syn4) were prepared to compare the abilities of those SNARE protein combinations to merge membranes. Both the tested combinations of SNARE proteins (SN23-Syn4-Vp2 and SN23-Syn4-Vp8) efficiently mediated membrane fusion, as indicated by the increase in the fluorescence intensity (Figure 3A), confirming that both of these SNARE complexes are active. When Vp2S or Vp8S was added to the reaction mixture, the membrane fusion mediated by SN23-Syn4-Vp2 or SN23-Syn4-Vp8 complexes was almost completely inhibited by both proteins (Figure 3B). The similarity between the activities of the two SNARE complexes containing each full-length soluble SNARE is consistent with the result that both β-hexosaminidase and histamine release were inhibited by both of those SNARE proteins (Figure 2D).

In contrast to the results described above, several peptides showed significantly different inhibitory activities against the SNARE complexes of SN23-Syn4-Vp2 and SN23-Syn4-Vp8 (Figure 3C). While most peptides inhibited membrane fusion by only 0–20%, the peptides SC23N, Syn4N, Vp2N, and Vp8N potently inhibited membrane fusion with specificities for different SNAREs. The peptides derived from the N-terminal region of the T-SNARE proteins SC23N and Syn4N inhibited membrane fusion by both the SN23-Syn4-Vp2 and SN23-Syn4-Vp8 complexes (Figures 3C,D). Vp2N efficiently inhibited the membrane fusion mediated by SN23-Syn4-Vp2 but not that mediated by SN23-Syn4-Vp8. In contrast, the inhibition of membrane fusion by Vp8N and Vp8M was specific to the SN23-Syn4-Vp8 complex. These results clearly indicated that the Vp2N and Vp8N peptides derived from the N-terminal region of the V-SNAREs VAMP2 and VAMP8 specifically inhibited the membrane fusion processes driven by VAMP2 and VAMP8, respectively.

Because the N-terminal nucleation step of SNARE complex formation is the rate-limiting step of SNARE-driven membrane fusion (39), the membrane fusion driven by the neuronal SNARE complex (SNAP-25-VAMP2-syntaxin 1a) was most efficiently inhibited when N-terminal binding flavonoids (cyanidin and delphinidin) (8, 40) or N-terminal-mimicking peptides were present (9). It is also possible that the efficiency and specificity of Vp2N and Vp8N against only their cognate SNARE complexes could be attributed to the rate-limiting N-terminal nucleation of the SNARE complexes comprising SN23-Syn4-Vp8 and SN23-Syn4-Vp2. The peptides Syn4N, SC23N, Vp2N, Vp8N, SN23C, and Vp8C were added to the reaction mixture containing the T-vesicle and each V-vesicle before or after preincubation at 4°C, which represents the condition in which the N-terminal SNARE proteins are partially zippered without any lipid mixing (38).

**Figure 4:**
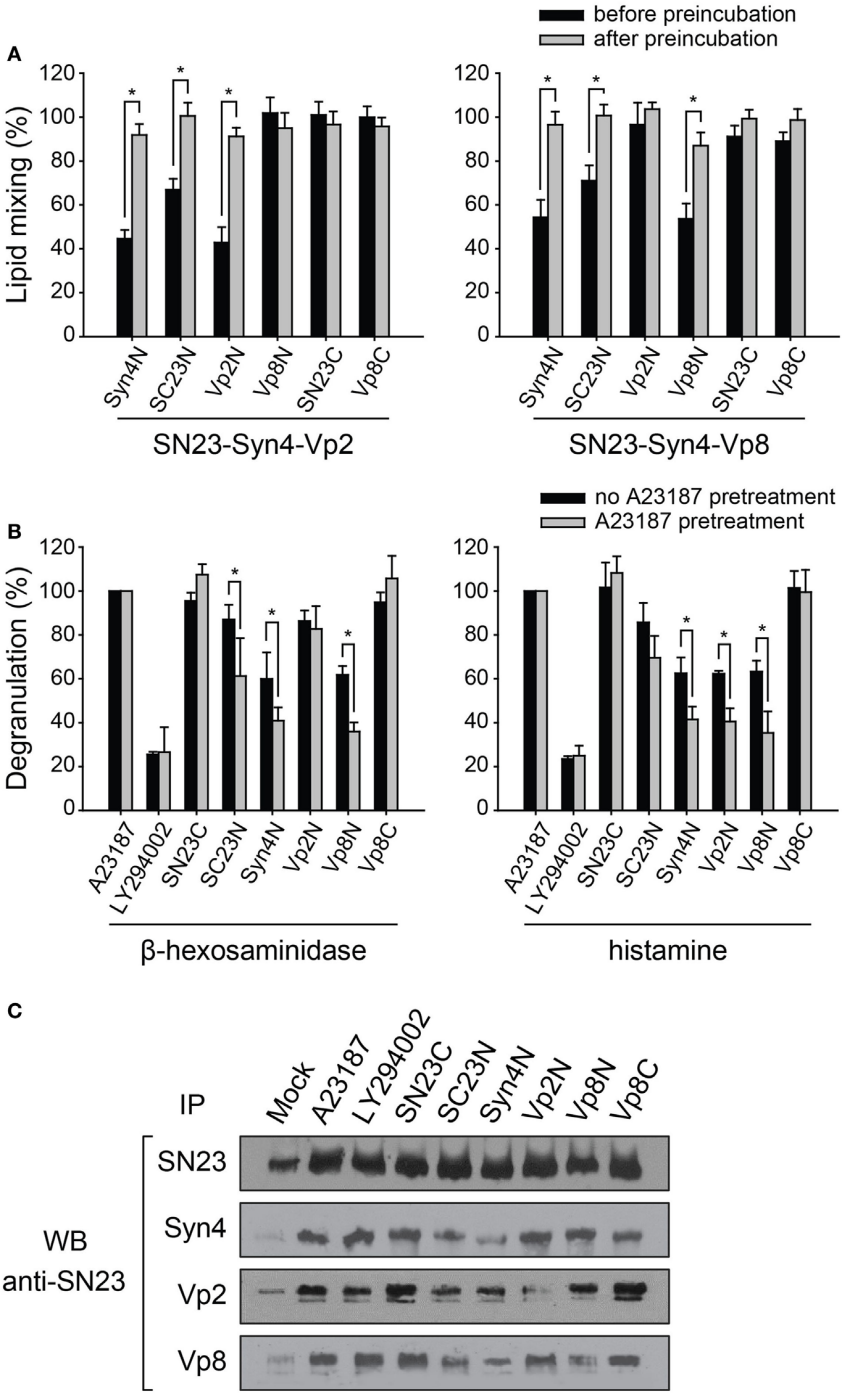
The inhibitory effect of synthetic peptides on the mast cell degranulation of distinct granule subsets. **(A)** Inhibitory effect of the N-terminal-mimicking peptides on membrane fusion dependent on the state of partial zippering of soluble N-ethylmaleimide-sensitive factor attachment protein receptor (SNARE) complex. The peptides were added before (black) or after the preincubation of T- and V-liposomes together at 4°C overnight (gray). Data are expressed as the mean ± SD. Significance: *p < 0.05. **(B)** The inhibitory effect of synthetic peptides on the degranulation of RBL-2H3 mast cells pretreated with A23187, which is an inducer of degranulation of mast cell without antigen-related FcεRI-crosslinking. After A23187 pretreatment, the mast cells were sensitized with DNP-IgE and then treated with protein transduction domain -conjugated peptides. The amounts of released granular components such as β-hexosaminidase and histamine were quantified by colorimetric biochemical assays. Data are expressed as the mean ± SD. Significance: *p < 0.05. **(C)** The extent of the formation of SNARE complexes in RBL-2H3 cells in the presence of inhibitory peptides was also examined by co-immunoprecipitation using an anti-SNAP-23 antibody.

When the peptides were added before preincubation, the inhibitory effects of the peptides were consistent with the above results, except that the inhibitory effects of the N-terminal-mimicking peptides increased [Figure 4A (gray bars) and Figure 3C]. The addition of SC23N and Syn4N derived from the T-SNARE proteins inhibited the membrane fusion induced by both SN23-Syn4-Vp2 and SN23-Syn4-Vp8 when the peptides were applied before preincubation. In contrast, Vp2N and Vp8N inhibited the membrane fusion driven only by their cognate SNARE complexes before preincubation. SN23C and Vp8C did not inhibit membrane fusion regardless of preincubation (Figure 4A). However, when the most effective peptides (Vp2N, Vp8N, Syn4N, and SC23N) were added after preincubation, their inhibitory activities significantly diminished (Figure 4A). Thus, the N-terminal-mimicking peptides inhibit membrane fusion by hindering the N-terminal nucleation step of SNARE complex formation.

In mast cells, docked vesicles tethered by the partial SNARE complexes on the plasma membrane allow the rapid release of contents of granules upon the crosslinking of the high-affinity IgE receptor on the cell surfaces (41). The inhibition of mast cell degranulation by the PTD-containing N-terminal peptides depending on the state of the SNARE complexes was assessed by pretreating the cells with the calcium ionophore A23187 (Figure S1A in Supplementary Material). A23187 has been known to induce mast cell degranulation without antigen-related FcεRI-crosslinking (Figure S1A in Supplementary Material). We first verified that the inhibitory effect of the peptides against β-hexosaminidase release was almost equal regardless of the stimulation method used (Figure S1B in Supplementary Material). LY294002, which inhibits FcεRI-mediated degranulation in mast cells due to its ability to control phosphoinositide 3-kinase (42), efficiently inhibited the degranulation of both β-hexosaminidase and histamine regardless of A23187 pretreatment. However, when the N-terminal-mimicking peptides (SC23N, Syn4N, Vp2N, and Vp8N) containing the N-terminal PTD sequence were added to the cell culture medium after A23187 pretreatment, they inhibited mast cell degranulation more efficiently than without pretreatment (Figure 4B). These results suggest that the N-terminal-mimicking peptides inhibited mast cell degranulation by inhibiting the N-terminal nucleation of SNARE complexes during SNARE recycling within the cells.

Vp2N did not inhibit the release of β-hexosaminidase, but it dramatically reduced the release of histamine, whereas Vp8N and Syn4N inhibited both histamine and β-hexosaminidase release (Figure 4B). SN23C and Vp8C did not inhibit degranulation regardless of whether the pretreatment with A23187 was performed. Because Vp2N and Vp8N inhibited the membrane fusion driven by the SN23-Syn4-Vp2 complex and the SN23-Syn4-Vp8 complex, respectively, these results are consistent with the previous finding that distinct secretory granules were regulated by different V-SNARE proteins.

We further investigated whether the peptide inhibitors could selectively inhibit SNARE complex formation in mast cells. The functional SNARE complex that formed after the activation of RBL-2H3 mast cells was identified by a co-immunoprecipitation analysis. The immunoprecipitation of Syn4, Vp2, and Vp8 in activated mast cell lysates followed by western blotting using an anti-SNAP-23 antibody suggested that the SN23-Syn4-Vp2 and SN23-Syn4-Vp8 complexes represent the major SNARE complexes in activated RBL-2H3 cells (Figure 4C). When the peptides were added to the medium after A23187 pretreatment, the SNARE complex formation was inhibited by the N-terminal-mimicking peptides, consistently with the above results, while LY294002 did not inhibit the formation of either the SN23-Syn4-Vp2 or SN23-Syn4-Vp8 complex.

The complexes containing both Syn4 and SN23, including the binary T-SNARE complex of Syn4-SN23 and the ternary SNARE complexes of SN23-Syn4-Vp2 and SN23-Syn4-Vp8, were the most dramatically reduced by Syn4N and SC23N. The amount of the SN23-Syn4-Vp8 complex was reduced by the Syn4N, SC23N, and Vp8N peptides, while the SN23-Syn4-Vp2 complex was reduced by Syn4N, SC23N, and Vp2N. In conclusion, the N-terminal-mimicking peptides reduce mast cell degranulation by inhibiting the nucleation step of SNARE complex formation in RBL-2H3 cells.

### Efficacy of Synthetic Peptides Against Atopic Dermatitis

**Figure 5:**
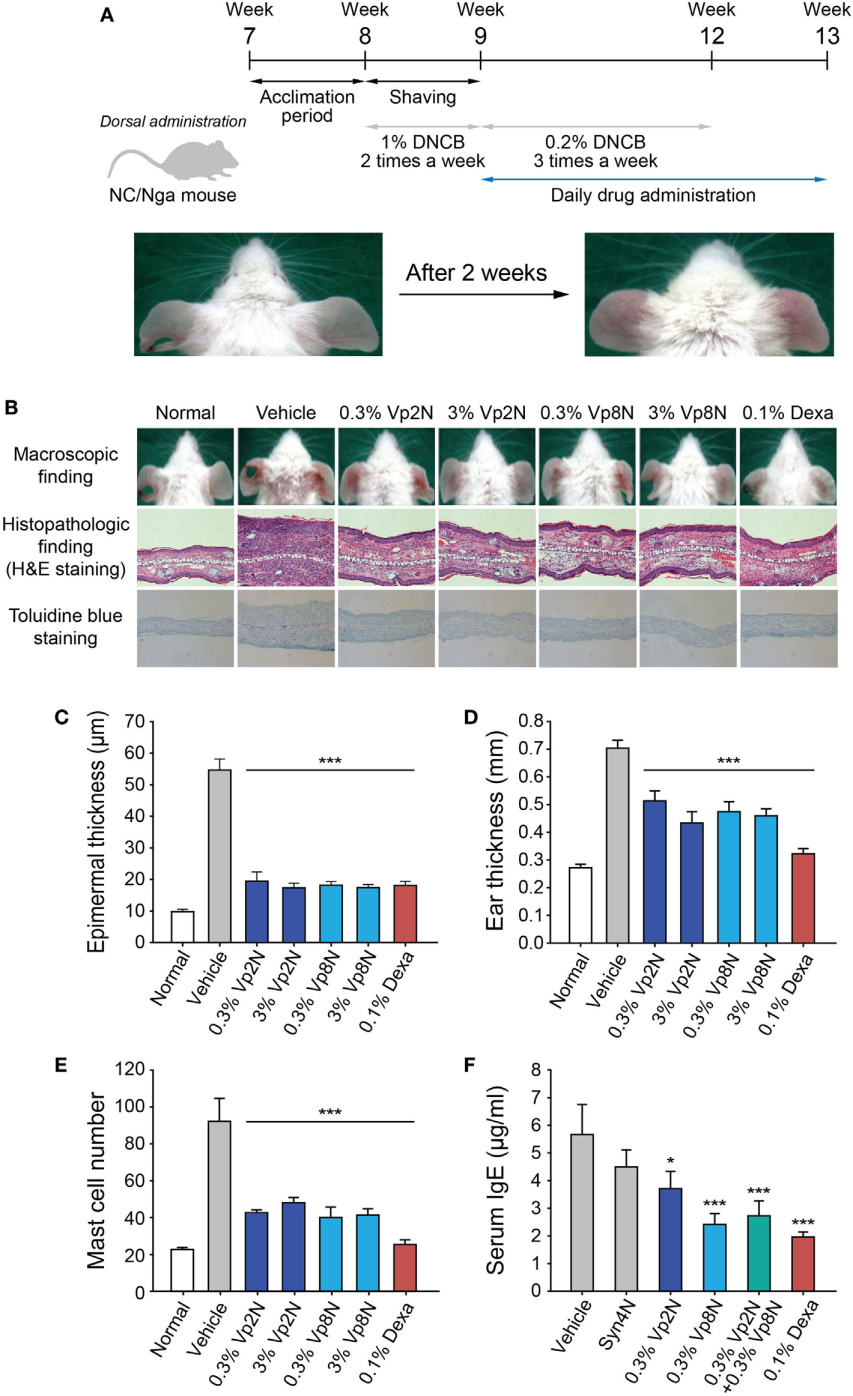
Efficacy of N-terminal-mimicking peptides for treating the DNCB-induced atopic dermatitis-like skin lesions of NC/Nga mice. **(A)** Experimental schedule for application of the experimental drugs to the dermatitis-like skin lesions induced in the NC/Nga mice. Following an initial sensitization step with 1% DNCB twice weekly for 1 week, the mice were dorsally treated with 0.2% DNCB thrice weekly for 3 weeks. The NC/Nga mice were daily treated with peptide drugs for 4 weeks starting from week 9. **(B)** The skin inflammation in the NC/Nga mice was reduced by the synthetic peptides. Macroscopic appearance of mice treated with synthetic peptides (upper panels) and histopathologic evaluation after the repeated application of synthetic peptides to the back skin of NC/Nga mice stained with hematoxylin and eosin (middle) or toluidine blue (lower). Epidermal thickness **(C)**, ear thickness **(D)**, the number of mast cells **(E)**, and the level of IgE **(F)** in serum were also evaluated. Dexamethasone (Dexa) was used as a positive control. Data are presented as the mean ± SD. *p < 0.05, ***p < 0.001 versus the control.

NC/Nga mice, which show atopic dermatitis-like skin lesions with the overexpression of type 2 helper cytokines and IgE, have been reported to be a suitable model for atopic dermatitis (43, 44). The skin lesions of NC/Nga mice are clinically and histologically similar to those of human atopic dermatitis when the mice are housed under conventional conditions. Skin lesions spontaneously appear on the face, neck, ears, and dorsal skin of NC/Nga mice at the age of 8 weeks. However, to induce a stable clinical atopic dermatitis-like skin disease, DNCB, which is an electrophilic and cytotoxic benzene derivative, was applied to the dorsal skin and the back of both ears of the NC/Nga mice in the present study (Figure 5A).

To examine the therapeutic effectiveness of the synthetic peptides in suppressing the skin lesions of NC/Nga mice, two selected synthetic peptides (Vp2N and Vp8N) or dexamethasone as a positive control were administered to mice with fully developed skin lesions (Figure 5A). Briefly, the mice were sensitized with 1% DNCB twice weekly after the acclimation period. Subsequently, 0.2% DNCB was administered to the mice thrice weekly for 3 weeks, and the peptides or dexamethasone were simultaneously administered daily for 4 weeks. After DNCB treatment, typical histological characteristics of atopic dermatitis skin lesions such as hyperkeratosis, partial epidermal defects, and a dense infiltration of leukocytes in the dermis were observed in the NC/Nga mice, whereas the control mice showed no histological abnormalities (Figure 5B). In hematoxylin and eosin-stained sections, atopic dermatitis features such as acanthosis, hypergranulosis, and hyperkeratosis were clearly alleviated to a similar extent in the NC/Nga mice that were treated with both the Vp2N and Vp8N peptides as in the dexamethasone-treated group (Figure 5B, middle panel). Additionally, the epidermal thickness was significantly reduced in both the peptide-treated groups as compared with the DNCB/vehicle-treated group (Figures 5B,C). The ear thickness of each mouse was measured to quantify the clinical severity of the DNCB-induced inflammation. The ear thickness was reduced by the peptide treatments, representing an inhibition of the inflammation. Dexamethasone recovered the ear thickness to the normal range (Figure 5D), but it has previously been reported to cause skin thinning as a side effect (45).

As previously reported (44), an immunohistochemical examination of the skin lesions showed an increase in the number of mast cells containing interleukin (IL)-4, which are necessary for IgE synthesis. Interestingly, the number of infiltrated mast cells significantly decreased in the dermis of mice treated with synthetic peptides as compared with the control mice in toluidine blue-stained sections (Figures 5B,E). The decrease in the number of infiltrated mast cells is likely to be responsible for the prevention of atopic dermatitis. It has already been shown that the plasma IgE levels of conventional NC/Nga mice become markedly elevated from the age of 8 weeks, correlating with the clinical severity of the dermatitis (43). Here, we found that the application of a synthetic peptide (Vp8N) significantly reduced the serum IgE levels of NC/Nga mice (Figure 5F). These results indicated that the administration of synthetic peptides macroscopically and histologically suppressed the atopic dermatitis skin lesions of NC/Nga mice in association with reduced serum IgE levels regardless of the differential selective regulatory effects of each peptide.

## Discussion

Botulinum neurotoxin is now widely applied in the cosmetic field and for the treatment of various neurological diseases such as strabismus, blepharospasm, hemifacial spasm, cervical dystonia, and many other indications (46). To realize new BoNT-like compounds for use in the cosmetic industry, there have been numerous attempts to mimic the efficacy of BoNT using peptides (9, 47) and plant-derived small molecules (4, 8, 10, 21, 48) by regulating neuronal SNARE complex formation. Acetyl hexapeptide-3 (Argireline^®^) is a commercialized synthetic peptide that is patterned from the N-terminal end of the SNAP-25 protein and inhibits SNARE complex formation as well as the release of catecholamine, adrenaline, and noradrenaline (49). The elongated form of Argireline^®^, acetyl octapeptide-3 (SNAP-8), has also been developed for reducing wrinkles in aging skin (50). In addition, the N-terminal-truncated SNAP-25 that is cleaved by BoNT/A or BoNT/E has been found to inhibit neurotransmitter release (16). Several peptides identified by library deconvolution that do not mimic the SNARE domain have also been shown to have inhibitory effects on the assembly of the SNARE complex (51–53).

Since the cell membrane fusion machinery has been clearly shown to be a versatile therapeutic target, an engineered BoNT that cleaves both SNAP-23 and SNAP-25 was previously developed (54). Although the therapeutic potential of that engineered BoNT has not been demonstrated yet, it is expected to have the potential for the treatment of human hypersecretion disorders such as atopic dermatitis, which is associated with a massive degranulation of mast cells. Thus, it was also plausible that a similar strategy used for the neuronal SNAREs can be applicable to mast cell SNAREs using peptides. Given that the ternary SNARE complexes of SN23-Syn4-Vp2 and SN23-Syn4-Vp8 are critically involved in the mast cell degranulation process (3, 19, 41), the inhibition of SNARE complex formation may provide an opportunity for therapeutic interventions against atopic dermatitis. Our challenge in the present study was to control the SNARE-mediated degranulation of mast cells and thereby regulate atopic dermatitis using synthetic peptides. The inhibitory effects of various peptides patterned after SNARE motifs against mast cell degranulation were investigated. *In vitro* experiments demonstrated a marked inhibition of the IgE-stimulated release of β-hexosaminidase and histamine by N-terminal-mimicking peptides through interference with Vp2- or Vp8-associated membrane fusion. This result is consistent with the previous results, where N-terminal-mimicking peptides of neuronal SNARE proteins were the most potent inhibitors of neurotransmitter release (9). It is because N-terminal nucleation is the rate-limiting step of SNARE complex formation. The peptides mimicking other regions of SNARE proteins showed very low inhibitory activity against SNARE-driven fusion and mast cell degranulation, consistently to those for neuronal SNARE complex. This low activity is attributed to the folding pathway of SNARE complex. C-terminal fragment of Vp2 (aa 49-96) bound to the binary acceptor complex can be readily removed by native Vp2 even without loss of speed (N- to C-Terminal SNARE Complex Assembly Promotes Rapid Membrane Fusion). Thus, 17-mer M- or C-peptides that bind to pre-formed binary complex are likely to be peeled off by native Vp2 and cannot inhibit full SNARE zippering. The only exception reported until now is myricetin which stops SNARE zippering in the middle (21). Because SNARE complexes have been shown to zipper in three distinct stages (52), the folding intermediate of ternary complex seems to expose a binding pocket near the ionic zeroth layer (21). The efficacy of N-terminal-mimicking peptides were confirmed in NC/Nga mice model with atopic dermatitis-like skin lesions. Although the detailed molecular mechanisms underlying the therapeutic and side effects of the peptides remain to be elucidated, our findings suggest that peptide inhibitors alleviate atopic dermatitis-like skin lesions in NC/Nga mice by targeting SNARE-mediated membrane fusion. Therefore, we propose that N-terminal-mimicking peptides may represent a promising topical therapeutic candidate for the control of atopic dermatitis.

Mast cells are multifunctional cells and play an important role in allergic and inflammatory responses. They are activated by a series of stimuli, releasing and producing inflammatory mediators (55, 56). The rat basophilic leukemia cells, RBL-2H3, are a tumor analog of mast cells, which can be activated by the IgE–antigen complex. In this study, RBL-2H3 cells were used to investigate the anti-allergic activity of SNARE-mimicking peptides. We note the limitations of RBL-2H3 cells for a model of mast cell mediator release because RBL-2H3 cells have been shown to have similarities to basophils in some aspects rather than other histamine-releasing cell types (57). However, RBL-2H3 cells are commonly employed as a prototypic model to study IgE-mediated degranulation of mast cells because they have similar granular content to mast cells, and the receptor-activated signaling pathways that result in degranulation is almost identical to those of primary mast cells (58). Furthermore, model human mast cells have limitations, too, because they are poorly reproducible and sometimes their biological properties are different from mast cells found *in vivo*. Thus, it is likely that RBL-2H3 cells hold good for assessing the allergenicity of antigens to humans unless purified and well-characterized mature primary cells are readily available (58).

The skin is our body’s important interface to the external environment. When skin barrier is disrupted, the first line of host defense does not work properly, causing immune dysfunction and inflammatory response. Atopic dermatitis is a common relapsing or chronic inflammatory disease of the skin caused by skin barrier dysfunction and cutaneous inflammation (59). Increased infiltration of T cells, macrophages, and mast cells can be observed in atopic dermatitis lesions. Mast cells are the key cells to mediate type I hypersensitivity reactions. Type I hypersensitivity is the term employed to describe IgE-mediated allergic disorders. It was demonstrated using animal models that mast cells are required for the development of atopic dermatitis-like skin lesion (60). Number of mast cells is increased in lesional atopic dermatitis skin. Mast cells secrete preformed and *de novo* synthesized proinflammatory and immunomodulatory mediators upon stimulation by IgE and antigen or other stimuli. In the acute phase of atopic dermatitis, invading allergens through damaged skin barrier may stimulate mast cells to release these inflammatory mediators. Furthermore, histamine released by mast cells can activate histamine receptor 1-expressing inflammatory macrophages via the histamine receptor on the surface of those cells (61). The mediators contribute to various aspects of systemic and local immune responses in atopic dermatitis (62, 63). Thus, alleviation of allergy provides a worthy therapeutic strategy for atopic dermatitis. Because both macrophages and mast cells in the skin are important in the initiation and progress of atopic dermatitis, topical anti-inflammatory agents like corticosteroids and antihistamine are commonly used for treatment of atopic dermatitis (64). As well as the repairing of skin barrier and avoidance of immunologic stimuli, systemic immunosuppressants can also be used as adjunctive treatments. As immune mediators and cytokines play a critical role in the initiation and exacerbation of atopic dermatitis blocking mast cell degranulation is crucial to abrogate the immediate hypersensitivity and to inhibit skin inflammation.

Finally, our results provide a new tool to test the roles of histamine and β-hexosaminidase in mediating the onset and progress of atopic dermatitis. Vp2N almost exclusively inhibited histamine release by interfering with the SN23-Syn4-Vp2 SNARE complex of type III granules, while Vp8N and Syn4N both inhibited the membrane fusion driven by the SN23-Syn4-Vp8 SNARE complex of type I and type II granules as well as the release of both β-hexosaminidase and histamine. It is interesting that the inhibition of the degranulation of only type III granules by Vp2N or simultaneously type I and II granules by Vp8N resulted in similar effects on atopic dermatitis, except for the serum IgE level (Figure 5). The effects of the selective regulation of distinct granule types deserve further investigation.

## Ethics Statement

Laboratory animal breeding management was based on the “Guide for the Care and Use of Laboratory Animals,” and all experiments were approved by the Institutional Animal Care and Use Committees of Gyeonggi Institute of Science & Technology.

## Author Contributions

YY and D-HK contributed conception and design of the study; YY, BK, J-MO, JH, and D-HK performed experiments; YJ, J-BP, J-MO, and JH performed the statistical analysis; YY and D-HK wrote the first draft of the manuscript; YY, BK, JC, and D-HK wrote sections of the manuscript. All authors contributed to manuscript revision, read and approved the submitted version.

## Conflict of Interest Statement

The authors declare that the research was conducted in the absence of any commercial or financial relationships that could be construed as a potential conflict of interest.
