# Supplementary material for: Soluble N-Ethylmaleimide-Sensitive Factor Attachment Protein Receptor-Derived Peptides for Regulation of Mast Cell Degranulation
Source: Front Immunol. 2018 Apr 11;9:725. doi: 10.3389/fimmu.2018.00725 (PMC5904360; doi:10.3389/fimmu.2018.00725)
Supplement: Supplementary file 1 [file Data_Sheet_1.DOCX]

Supplementary Material

SNARE-derived Peptides for the Treatment of Atopic Dermatitis

Yoosoo Yang^a,b,1^, Byoungjae Kong^c,d,1^, Younghoon Jung^c,d^, Joon-Bum Park^c,d^, Jung-Mi Oh^c^, Jaesung Hwang^e^, Jae-Yeol Cho^c,d^ and Dae-Hyuk Kweon^c,d,*^

*** Correspondence:** Dr. Dae-Hyuk Kweon: [dhkweon@skku.edu](mailto:dhkweon@skku.edu)

# Supplementary Figures

**
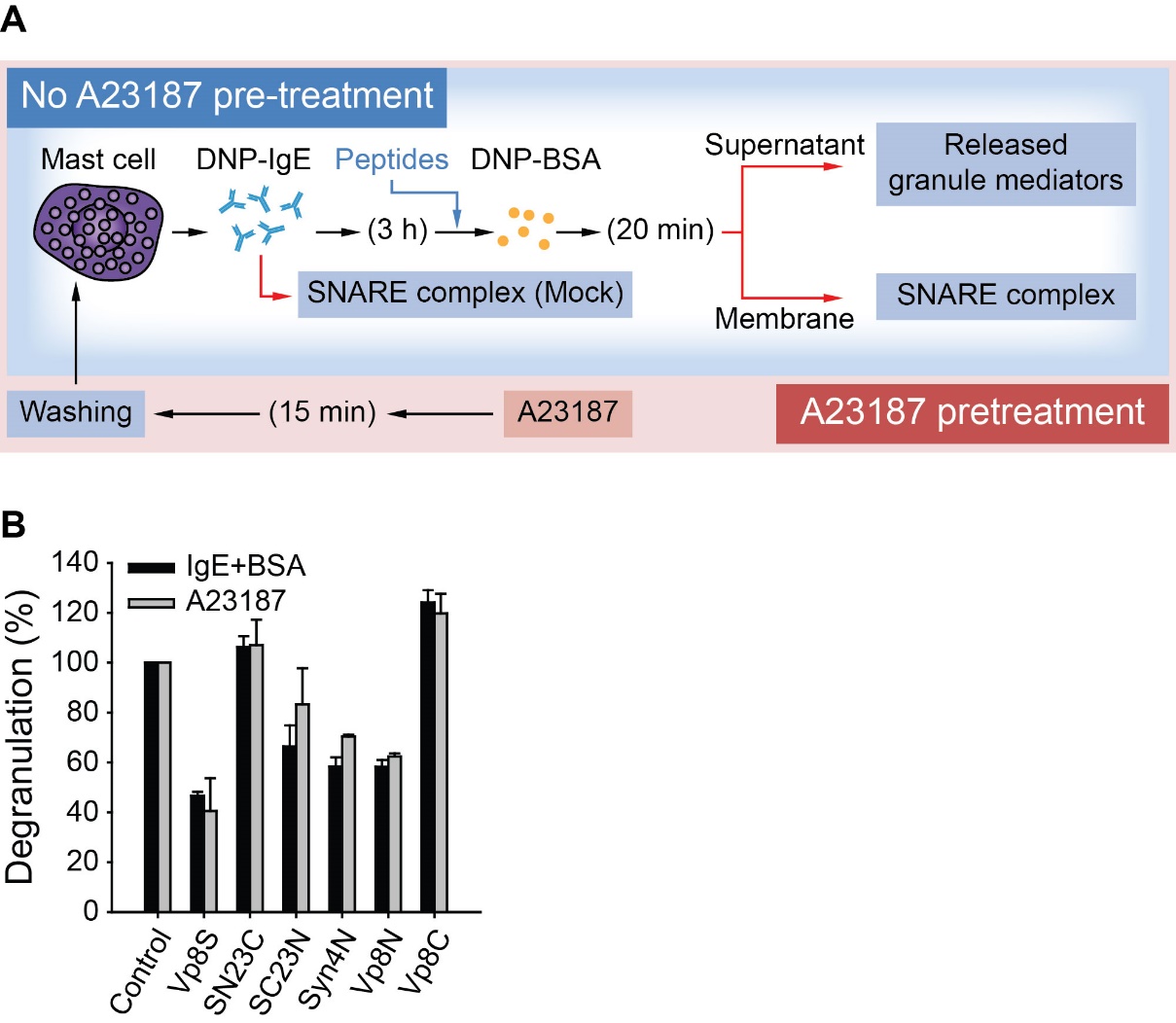
**

**Supplementary Figure 1.** **Experimental scheme to evaluate the efficacy of inhibitory efficacy of synthetic peptides on the degranulation of RBL-2H3 mast cells with or without pretreatment with A23187.** (**A**) Experimental schemes. Red box, A23187 pretreatment; blue box, no pretreatment group. (**B**) The inhibitory effects of the synthetic peptides against the release of β-hexosaminidase from mast cells stimulated with IgE/antigen (black) or the calcium ionophore A23187 (gray).
